# Integrating Multi-Type Component Determination and Anti-Oxidant/-Inflammatory Assay to Evaluate the Impact of Pre-Molting Washing on the Quality and Bioactivity of Cicadae Periostracum

**DOI:** 10.3390/molecules27227683

**Published:** 2022-11-08

**Authors:** Meng-Fei Guo, Huan-Huan Zhang, Ping Zhong, Jin-Di Xu, Shan-Shan Zhou, Fang Long, Ming Kong, Qian Mao, Song-Lin Li

**Affiliations:** 1 Department of Pharmaceutical Analysis, Affiliated Hospital of Integrated Traditional Chinese and Western Medicine, Nanjing University of Chinese Medicine, Nanjing 210028, China; 2 Department of Metabolomics, Jiangsu Province Academy of Traditional Chinese Medicine, Nanjing 210028, China

**Keywords:** Cicadae Periostracum, primary processing, pre/post-molting washing, quality, bioactivity

## Abstract

Cicadae Periostracum (CP) is a traditional Chinese medicinal herb derived from the slough that is molted from the nymph of the insect *Cryptotympana pustulata* Fabricius. Washing with water to remove residual silt is a primary processing method of CP that is recommended by the Chinese Pharmacopoeia, but how washing methods affect the quality and bioactivity of CP is unknown. In this study, the quality and bioactivity of non-washed CP (CP-NW), post-molting-washed CP (CP-WAT), and pre-molting-washed CP (CP-WBT) were comparatively investigated. The quality of these CP samples was evaluated in terms of the UPLC-QTOF-MS/MS-based chemical profiling and semi-quantification of 39 *N*-acetyldopamine oligomers (belonging to six chemical types), the HPLC-UV-based quantification of 17 amino acids, the ICP-MS-based quantification of four heavy metals, and the contents of ash; the bioactivities of the samples were compared regarding their anti-oxidant and anti-inflammatory activities. It was found that, compared with CP-NW, both CP-WBT and CP-WAT had significantly lower contents of ash and heavy metals. Moreover, compared with CP-WAT, CP-WBT contained lower levels of total ash, acid-insoluble ash, and heavy metals and higher contents of *N*-acetyldopamine oligomers and amino acids. It also had enhanced anti-oxidant and anti-inflammatory activities. A Spearman’s correlation analysis found that the contents of *N*-acetyldopamine oligomers and free amino acids were positively correlated with the anti-oxidant/-inflammatory activities of CP. All these results suggest that pre-molting washing can not only remove the residual silt but can also avoid the loss of the bioactive components and assure higher bioactivities. It is concluded that pre-molting washing could enhance the quality and bioactivity of CP and should be a superior alternative method for the primary processing of qualified CP.

## 1. Introduction

Cicadae Periostracum (CP), derived from the slough that is molted from the nymph of the insect *Cryptotympana pustulata* Fabricius [1], is a widely-used traditional Chinese medicinal herb for relieving fevers, soothing throats, expelling rashes, and relieving spasms [2,3]. It has been utilized not only as a common drug for clinical formulations but also as one of the main constituted raw materials of many famous traditional Chinese patent medicines, such as Huangshi-Xiangsheng-Wan pills for treating hoarse voices and sore throats, Suhuang-Zhike-Jiaonang capsules for curing cough and throat itching, and Toujie-Quwen-Keli granules for relieving the symptoms of coronavirus disease 2019 (COVID-19) [4,5,6].

Traditionally, CP (the slough of *C. pustulata*) is collected by farmers from trees, upon the different parts on which the nymphs of *C. pustulata* climbed from the ground and molted during the times from 7 p.m. to the next 6 a.m. in summer [7]. However, this collection method has two drawbacks. First, the sites that the nymphs climbed upon and molted are often relatively high and scattered on the trees, which makes the slough collection very laborious and time-consuming. Second, as specified in the Chinese Pharmacopoeia (2020 Version), the collected slough should be washed with water to remove the residual silt and obtain the qualified CP (which we refer to as post-molting-washed slough, CP-WAT) [1], but this primary processing method may cause the loss of some water-soluble bioactive constituents of slough and consequently affect the quality or bioactivity of the CP. Recently, a new strategy for efficiently collecting and performing the primary processing of slough of *C. pustulata* was practically proposed: first, one-sided smooth and adhesive tape (about five centimeters wide) was fixed around the tree trunk (about one meter above the ground) to prevent the nymphs of *C. pustulata* from climbing upon the higher parts of the trees; second, the nymphs were efficiently collected, put into water to make them dormant, and then collectively washed with water; third, the washed nymphs were placed into a net cage to wait for their molting to obtain the slough (which we refer to as pre-molting-washed slough, CP-WBT). This new strategy actually improves the efficiency of slough collection. However, how this method affects the quality and bioactivity of CP is still unknown.

It has been reported that the extracts of CP have several bioactivities, including anti-oxidant, anti-inflammatory, spasmolytic, and anti-asthma, etc. [8,9,10]. Our previous studies found that *N*-acetyldopamine oligomers were the main components that might be responsible for the activities of CP [11,12]. Moreover, amino acids have been reported as another type of bioactive component of CP [13]. In addition, the residual silt and heavy metals have are considered to be the major factors that affect the quality of CP [14,15].

In this study, the quality of CP samples that derived from pre-molting-washed slough (CP-WBT), post-molting-washed slough (CP-WAT), and non-washed slough (CP-NW) were systematically evaluated by ultra-high-performance liquid chromatography–quadrupole time-of-flight mass spectrometry (UPLC-QTOF-MS/MS)-based chemical profiling and the semi-quantitative determination of 39 *N*-acetyldopamine oligomers (belonging to six chemical types), the high-performance liquid chromatography-ultraviolet (HPLC-UV)-based quantification of 17 amino acids, the inductively coupled plasma mass spectrometry (ICP-MS)-based quantification of four heavy metals (As, Hg, Pb, and Cd), and the contents of total ash and acid-insoluble ash. Furthermore, the anti-oxidant and anti-inflammatory activities of the three kinds of CP samples were also comparatively investigated. A Spearman’s correlation analysis was used to understand the relationships between the contents of the multi-type components and the anti-oxidant/-inflammatory activities.

## 2. Results and Discussion

### 2.1. Comparison of Total Ash and Acid-Insoluble Ash in Three Kinds of CP Samples

**Figure 1:**
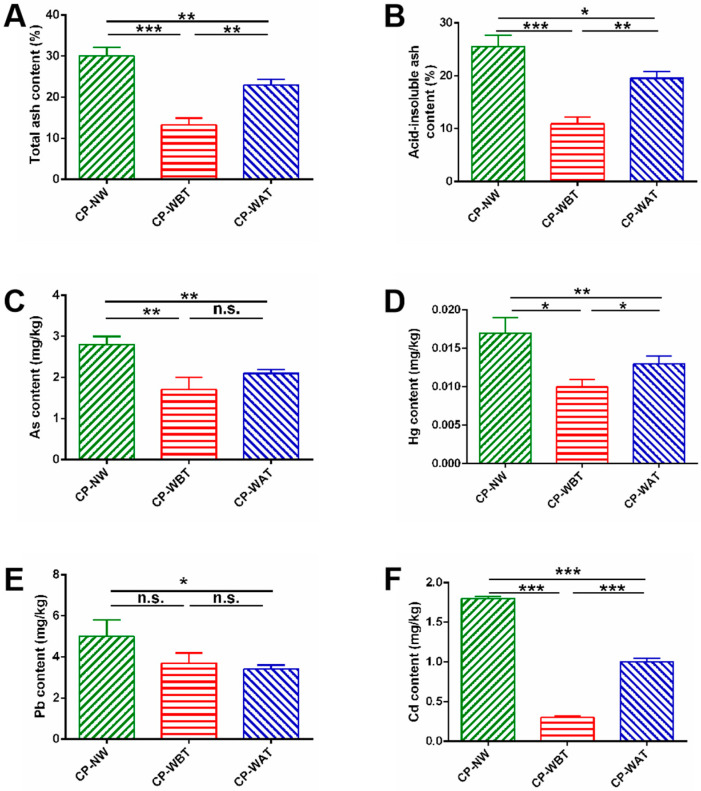
The contents of ash and heavy metals in three kinds of CP samples. (**A**) total ash, (**B**) acid-insoluble ash, (**C**) As, (**D**) Hg, (**E**) Pb, and (**F**) Cd. Data represent means ± SD, *n* = 3. *** *p <* 0.001, ** *p <* 0.01, * *p* < 0.05.

The contents of total ash and acid-insoluble ash in CP-WBT, CP-WAT, and CP-NW are shown in Figure 1A,B. It was found that CP-NW had the highest contents of both total ash and acid-insoluble ash (30.01% and 25.52%, respectively), which had significant differences from those in CP-WBT (*p* < 0.001) and CP-WAT (*p* < 0.05), suggesting that water washing can dramatically decrease the contents of ash in CP samples. Moreover, obvious differences were observed in the contents of total ash and acid-insoluble ash between CP-WBT and CP-WAT (*p* < 0.01). The total ash and acid-insoluble ash in CP-WBT were 9.73% and 8.60% lower than those in CP-WAT, respectively. The reason for these results may be that water washing can remove the residual silt on the surface of the slough, so the contents of total ash and acid-insoluble ash in CP-WBT and CP-WAT were lower than those of CP-NW. However, post-molting washing could cause more contact of the medial side and lateral side of slough with the silt-containing water than the pre-molting washing in which only the lateral side is contactable with silt-containing water. Consequently, the CP-WAT had more residual silt than CP-WBT. It is inferred that pre-molting washing may be a superior alternative primary process to lower the ash residue in CP.

### 2.2. Comparison of Four Heavy Metals in Three Kinds of CP Samples

As depicted in Figure 1C–F, the contents of As, Hg, Pb, and Cd in CP-WBT and CP-WAT were obviously lower than those in CP-NW. Furthermore, the contents of Hg and Cd in CP-WBT were significantly lower than those in CP-WAT, and the contents of As, Hg, Pb, and Cd in CP-WBT were low enough to meet the ‘Green Industry Standard for Import and Export of Medicinal Plants and Preparations’ (As ≤ 2.0 mg/kg, Hg ≤ 0.2 mg/kg, Pb ≤ 5 mg/kg, and Cd ≤ 0.3 mg/kg) (China, 2001) [16]. It is well-accepted that the contents of heavy metals in traditional Chinese medicines (TCMs) that exceed the standard might pose threats to human health [17,18,19]. Hence, it is of great importance to control the residual heavy metals in TCMs. Our results proved that the washing process could decrease the contents of toxic heavy metals (As, Hg, Pb, and Cd) in CP samples. The newly proposed pre-molting washing method might be a superior alternative process to lower the contents of residual heavy metals in CP.

### 2.3. Qualitative and Quantitative Comparison of N-acetyldopamine Oligomers in Three Kinds of CP Samples 

**Figure 2:**
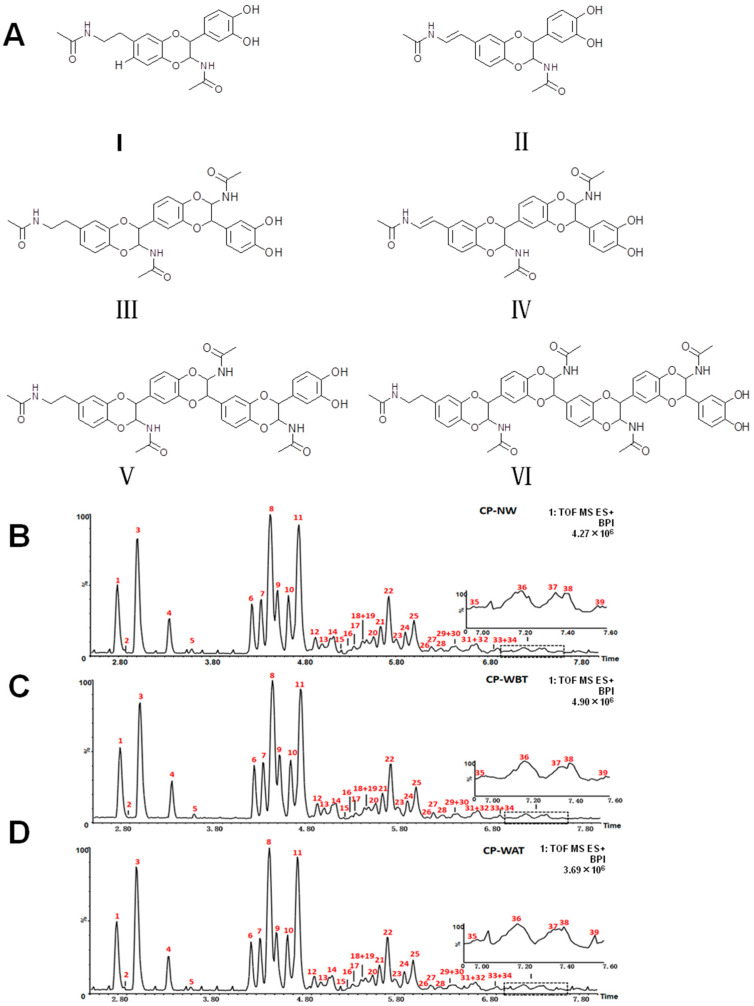
Chemical structures of different types of *N*-acetyldopamine oligomers and UPLC-QTOF-MS BPI chromatograms of three kinds of CP samples in positive ion mode. (**A**) (I) dimers, (II) side-chain isomer of dimers, (III) trimers, (IV) side-chain isomer of trimers, (V) tetramers, (VI) pentamers; (**B**) CP-NW; (**C**) CP-WBT; (**D**) CP-WAT.

As shown in Figure 2, the chemical profiles of the three kinds of CP samples were consistent with each other. A total of 39 *N*-acetyldopamine oligomers were identified from the three kinds of CP samples, including 4 *N*-acetyldopamine dimers, 1 side-chain isomer of dimers, 8 *N*-acetyldopamine trimers, 7 side-chain isomers of trimers, 10 *N*-acetyldopamine tetramers, and 9 *N*-acetyldopamine pentamers [11,12,20,21,22]. Five more side-chain isomers of trimers were tentatively identified compared with our previous study [11,12]. The structural information of the 39 *N*-acetyldopamine oligomers is summarized in Table 1.

**Figure 3:**
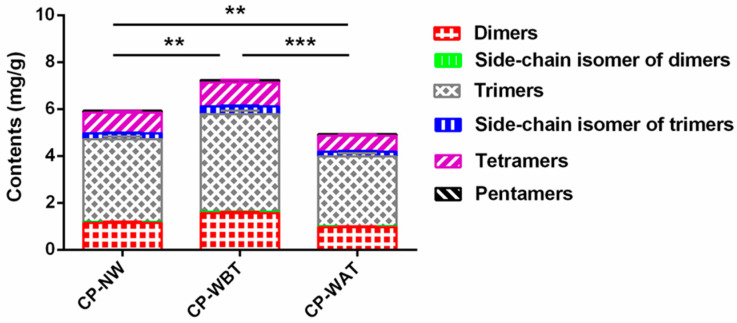
Contents of *N*-acetyldopamine oligomers in three kinds of CP samples. Data represent means ± SD, *n* = 3. *** p < 0.001, ** p < 0.01.

Due to similar parent nuclei, six types of *N*-acetyldopamine oligomers, including *N*-acetyldopamine dimers, a side-chain isomer of dimers, *N*-acetyldopamine trimers, a side-chain isomer of trimers, *N*-acetyldopamine tetramers, and *N*-acetyldopamine pentamers, were semi-quantified using *N*-acetyldopamine dimer A as a reference compound, referring to our previous method [11]. As shown in Figure 3, the total content of *N*-acetyldopamine oligomers in CP-WBT (7.24 ± 0.36 mg/g) was significantly higher than those in CP-NW (5.93 ± 0.26 mg/g) and CP-WAT (4.93 ± 0.23 mg/g), which were 22.09% and 46.86% lower, respectively, whereas the total content of *N*-acetyldopamine oligomers in CP-WAT was significantly lower than that in CP-NW, which was 16.86% higher, suggesting that pre-molting washing could increase the contents of *N*-acetyldopamine oligomers, whereas post-molting washing could decrease that in the CP samples. It was reported that *N*-acetyldopamine oligomers could stabilize an insect’s cuticles by their incorporation into the cuticular proteins during sclerotization [23,24]. In light of the above results, it is proposed that pre-molting washing may stimulate the production of *N*-acetyldopamine oligomers in the nymphs of *C. pustulata*, thus resulting in the increased contents of *N*-acetyldopamine oligomers in CP-WBT. Another reason for the results is that pre-molting washing can remove the residual silt on the surface of the nymphs and consequently increase the relative contents of *N*-acetyldopamine oligomers, whereas post-molting washing may cause a greater loss of *N*-acetyldopamine oligomers owing to the greater contact of both the medial side and lateral side of the slough with water compared to pre-molting washing in which only the lateral side of the slough was contactable with water, though the residual silt on the lateral side was also removed during washing. As *N*-acetyldopamine oligomers are major bioactive components of CP [8,11,12], it is concluded that pre-molting washing is a superior primary processing method to improve the quality of CP based on the quantitative results of *N*-acetyldopamine oligomers.

### 2.4. Qualitative and Quantitative Comparisons of Amino Acids in Three Kinds of CP Samples 

**Figure 4:**
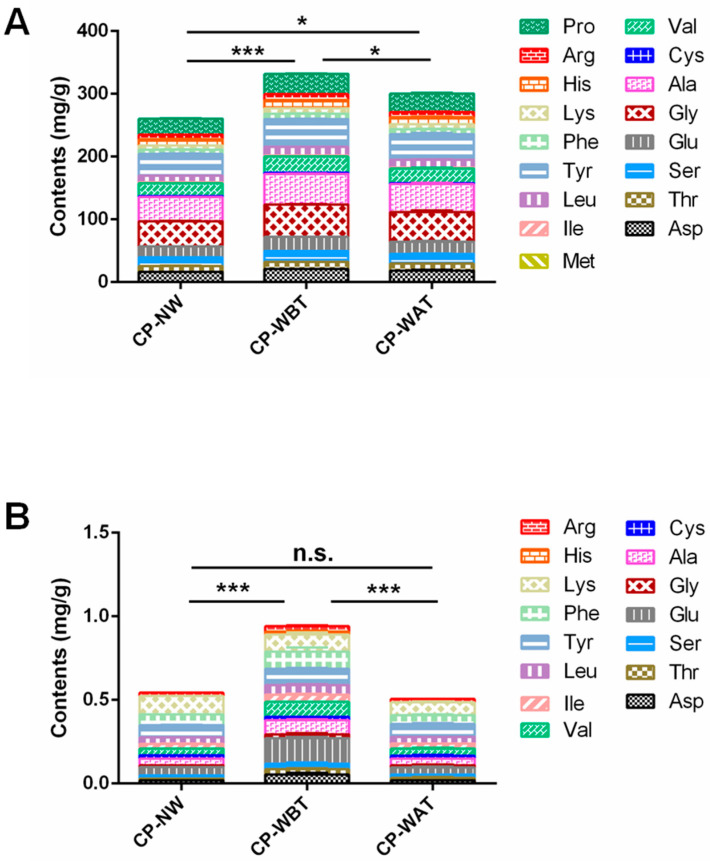
Total contents of amino acids in three kinds of CP samples. (**A**) hydrolyzed amino acids (HAA); (**B**) free amino acids (FAA). Data represent means ± SD, *n* = 3. *** p < 0.001, * p < 0.05.

For the qualitative and quantitative comparisons of amino acids in the three kinds of CP samples, 17 amino acids were determined. As shown in Figure 4A, the compositions of amino acids in CP-WBT, CP-WAT, and CP-NW were similar. However, the total contents of hydrolyzed amino acids (HAA) in CP-NW (260.51 ± 3.37 mg/g) were significantly lower than those in CP-WBT (331.95 ± 1.88 mg/g, *p* < 0.01) and CP-WAT (300.39 ± 14.72 mg/g, *p* < 0.05). Furthermore, the total content of HAA in CP-WBT was significantly higher (10.51% higher) than that in CP-WAT (*p* < 0.05). On the other hand, the total content of free amino acids (FAA) in CP-WBT (0.94 ± 0.02 mg/g) was significantly higher than those of CP-WAT (0.51 ± 0.01 mg/g, *p* < 0.001) and CP-NW (0.54 ± 0.02 mg/g, *p* < 0.001), which were 84.31% and 74.07% lower, respectively, while no significant difference in FAA contents was found between CP-NW and CP-WAT (Figure 4B). The above results imply that pre-molting washing could increase the contents of both HAA and FAA in CP, while post-molting washing could only increase the contents of HAA. The explanation of these results is similar to that for the *N*-acetyldopamine oligomers content, i.e., pre-molting washing can remove the residual silt on the surfaces of the nymphs and consequently increase the relative contents of HAA and FAA., whereas post-molting washing leads to a greater loss of amino acids, in particular FAA, owing to the water solubility of FAA and the greater contact of both the medial side and lateral side of the slough with water. Therefore, pre-molting washing is superior to post-molting washing for the primary processing of CP when taking into account the retention of amino acids, in particular FAA.

### 2.5. Comparison of DPPH and ABTS Radical Scavenging Rates of Three Kinds of CP Samples 

**Figure 5:**
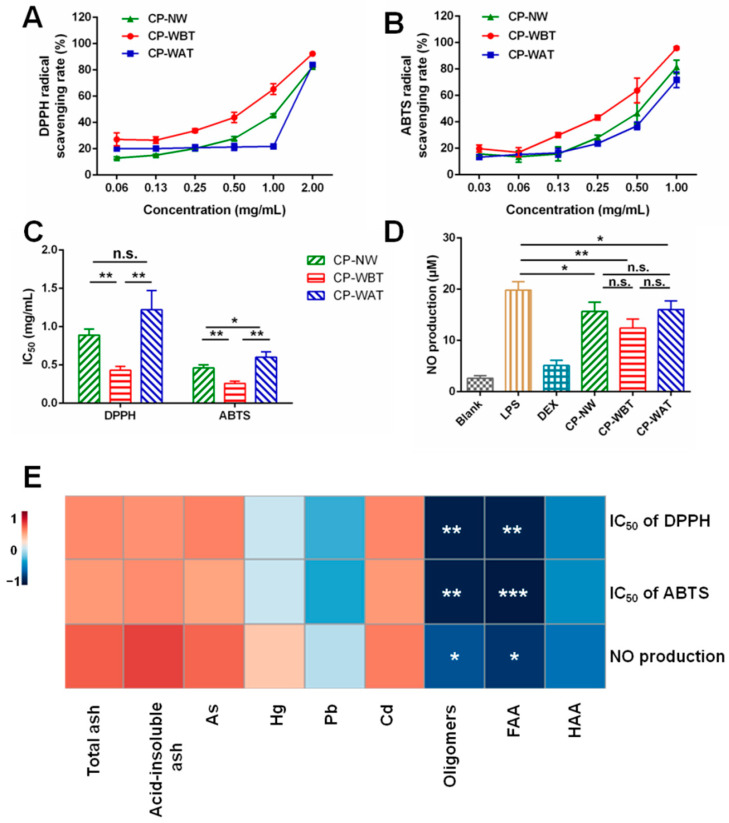
Anti-oxidant/-inflammatory activities and correlations with contents of multi-components of three kinds of CP samples. (**A**) DPPH radical scavenging rate; (**B**) ABTS radical scavenging rate; (**C**) Values of IC_50_; (**D**) NO production in RAW264.7 cells; (**E**) Spearman’s correlations between the contents of multi-type components and anti-oxidant/-inflammatory activities. Data represent means ± SD, *n* = 3. *** p < 0.001, ** p < 0.01, * p < 0.05.

As depicted in Figure 5A, the 2,2′-diphenyl-1-picrylhydrazyl (DPPH) free radical scavenging effect of each group was gradually enhanced with increases in the CP extract concentration, showing a positive correlation trend. Compared with CP-NW, CP-WBT had a significantly lower IC_50_ value (*p* < 0.01), but no significant difference in the IC_50_ values was found between CP-WAT and CP-NW (Figure 5C). Additionally, the 2,2’-azino-bis (3-ethylbenzo-thiazoline-6-sulfonic acid) diammonium salt (ABTS) radical scavenging rate presented a similar trend to DPPH, as shown in Figure 5B. Compared with CP-NW, CP-WBT showed a significantly lower IC_50_ value (*p* < 0.01), whereas CP-WAT had a significantly higher IC_50_ value (*p* < 0.05). The above results indicate that pre-molting washing significantly enhanced the anti-oxidant activity of CP, whereas post-molting washing weakened the anti-oxidant effect of CP. It was reported that *N*-acetyldopamine dimers and some amino acids such as lysine and arginine have anti-oxidant activities [8,25,26]. The varied anti-oxidant capacities of the three kinds of CP samples may be attributed to the relatively higher contents of *N*-acetyldopamine oligomers and amino acids in CP-WBT and the relatively lower contents of *N*-acetyldopamine oligomers in CP-WAT. Hence, pre-molting washing is superior to post-molting washing for the primary processing of CP when the anti-oxidant activity is concerned.

### 2.6. Comparison of Nitric Oxide (NO) Production among Three Kinds of CP Samples 

Excessive NO production leads to a series of pathological and physiological reactions when an inflammatory response occurs [27,28]. Inhibiting NO production can reduce damage during an inflammatory response [29]. Lipopolysaccharide (LPS)-stimulated RAW264.7 macrophages were used to establish a classic in vitro model to evaluate the anti-inflammatory effect of CP. As shown in Figure 5D, compared with the LPS group, CP-WBT, CP-WAT, and CP-NW could all significantly reduce the production of NO, with CP-WBT having more significant effects. This demonstrated that CP-WBT had the most significant anti-inflammatory effect among the three kinds of CP samples. It was reported that *N*-acetyldopamine dimers from CP had anti-inflammatory activities [8], and the anti-inflammatory bioactivity of *N*-acetyldopamine dimers involves suppressing the expression of the inflammatory mediators iNOs and COX-2, including cytokines, via the NF-κB signaling pathway in LPS-induced macrophages. Moreover, the *N*-acetyldopamine dimers effectively inhibited cathepsin C activity at the cellular level [30]. Some amino acids also possessed anti-inflammatory activities [31,32]. The most efficient NO production inhibition of CP-WBT may be ascribed to its higher contents of *N*-acetyldopamine oligomers and amino acids among the three kinds of CP samples. Therefore, pre-molting washing is superior to post-molting washing for the primary processing of CP when the anti-inflammatory effect is concerned.

### 2.7. Correlations between Contents of Multi-Type Components and Anti-Oxidant/-Inflammatory Activities

As shown in Figure 5E, the contents of *N*-acetyldopamine oligomers had significant correlations with the IC_50_ values of DPPH and ABTS, where the lower the IC_50_ value, the stronger the anti-oxidant capacity, indicating that the contents of *N*-acetyldopamine oligomers were positively correlated with anti-oxidant activities. In addition, the contents of oligomers showed significant correlations with NO production, where the lower the production of NO, the stronger the anti-inflammatory capacity, suggesting that the contents of *N*-acetyldopamine oligomers were positively correlated with the anti-inflammatory activities. Similarly, the contents of FAA were also positively correlated with the anti-oxidant and anti-inflammatory activities. These results further verified the reason why CP-WBT showed higher anti-oxidant/-inflammatory activities than the other two kinds of CP samples in this study.

## 3. Materials and Methods

### 3.1. Reagents and Chemicals

LC-MS-grade methanol and acetonitrile were sourced from Merck Co., Ltd. (Darmstadt, Germany). Formic acid (FA) was obtained from ROE Scientific. Inc. (Newark, DE, USA). Signal–element calibration standard solutions of arsenic (As), mercury (Hg), lead (Pb), and cadmium (Cd) (1000 μg/mL) were bought from Guobiao Testing Certification Co., Ltd. (Beijing, China). Amino acid mixture standard solutions were supplied by Wako Pure Chemical Industries, Ltd. (Osaka, Japan).

Fetal bovine serum (FBS) was purchased from Evergreen Bio (Hangzhou, China). Dulbecco’s Modified Eagle Medium (DMEM) was bought from Keygen Bio (Nanjing, China). DPPH and a Griess reagent assay were obtained from Aladdin Biochemical Technology Co., Ltd. (Shanghai, China). ABTS was obtained from Sigma Chemical Co. (St. Louis, MO, USA). LPS was purchased from Biosharp Co., Ltd. (Hefei, China). Ultrapure water was supplied by a Milli-Q water purification system (Elix 5, Millipore, Bedford, MA, USA). The other employed reagents and chemicals were of analytical grade.

The reference compounds of *N*-acetyldopamine dimer A (2*R*, 3*S*)-2-(3′,4′-dihydroxyphenyl)-3-acetylamino-7-(*N*-acetyl-2′’-aminoethyl)-1,4-benzodioxane) and *N*-acetyldopamine dimer B (2*R*, 3*S*)-2-(3′,4′-dihydroxyphenyl)-3-acetylamino-6-(*N*-acetyl-2′’-aminoethyl)-1,4-benzodioxane) were isolated from CP by a silica column, an octadecyl silane column, and preparative reverse-phase high-performance liquid chromatography (RP-HPLC) in our laboratory. The chemical structures of the two compounds were identified by UV, MS, ^1^H nuclear magnetic resonance (NMR), and ^13^C NMR. The purity of each reference compound was >98% by high-performance liquid chromatography coupled with a photodiode array detector (HPLC-DAD) [11].

CP samples were collected from Pei County, Xuzhou City, Jiangsu Province of China, and were authenticated as the slough of *C. pustulata* by Prof. Song-Lin Li by referring to the monograph documented in the Chinese Pharmacopoeia (Part I, 2020 Version). The samples were divided into three groups (CP-NW, CP-WBT, and CP-WAT) and three batches per group. Voucher specimens (JSPACM-66-50-1~JSPACM-66-50-3, JSPACM-66-51-1~JSPACM-66-51-3, and JSPACM-66-52-1~JSPACM-66-52-3) were deposited at the Department of Metabolomics of Jiangsu Province Academy of Traditional Chinese Medicine.

### 3.2. Sample Collection and Primary Processing 

CP-WBT samples: First, one-side smooth and adhesive tape (about 5 cm wide) was fixed around the tree trunk (about 1 m above the ground) to prevent the nymphs of *C. pustulata* from climbing upon the higher parts of the tree. Second, the nymphs were collected during the time from 7 p.m. to 9 p.m., and the collected nymphs were put into a container filled with clean water to make the nymphs become dormant. Third, the dormant nymphs were gently washed with water three times (about 20 mL of water for each nymph), and the washed nymphs were put in a net cage to wait for the nymphs to molt on the net and obtain the pre-molting-washed slough.

CP-WAT samples: The nymphs freely climbed and molted upon the trees, and the collected sloughs were washed with water three times (about 20 mL of water for each slough) to obtain the post-molting-washed slough.

CP-NW samples: The nymphs freely climbed and molted upon the trees, and the slough was collected to obtain non-washed slough.

All CP-WBT, CP-WAT, and CP-NW samples were dried with ventilation.

### 3.3. Determination of Total Ash and Acid-Insoluble Ash

The total ash content was determined by the method recommended by the Chinese Pharmacopoeia (Part IV, 2020 Version). In brief, 3 g of the CP powder (through a 24-mesh sieve) was weighed and put in a crucible that had been dried to a constant weight. The temperature was gradually increased to 550 °C, and the sample was slowly heated to carbonization [33]. After 6 h, the sample was fully ashed and reached a constant weight. The total ash content (%) was calculated according to the residue weight of the sample.

For the acid-insoluble ash content determination, 10 mL of diluted hydrochloric acid was added to the ash above in the crucible. Then, the crucible was covered with a watch glass and was heated in a water bath for 10 min. The watch glass was rinsed with 5 mL of hot water, and the washing liquid was incorporated into the crucible. The residue in the crucible was filtered with ash-free filter paper and washed until the washing liquid showed no chloride reaction. Both the filter residue and filter paper were transferred to the same crucible. They were dried and burned to a constant weight. The acid-insoluble ash content (%) was calculated according to the residue weight of the sample in the crucible.

### 3.4. Determination of Common Heavy Metals

The contents of As, Hg, Pb, and Cd in the CP samples were measured using ICP-MS (NexION 2000, PerkinElmer, Norwalk, CT, USA). In brief, each powdered sample (0.1 g) was directly weighed into teflon digestion vessels and then added to 2 mL of HNO_3_. After 20 min of pre-digestion, the closed vessel was digested in a microwave digestion system (Ethos Touch Control, Milestone Ltd., Bergamo, Italy) [34]. The digestion program was as follows: (1) 600 W at 160 °C for 5 min, (2) 800 W at 210 °C for 3 min, and (3) 600 W at 210 °C for 15 min. After the digestion was finished, the sample was transferred to a 25 mL volumetric flask. Then, the digestion tank was washed with ultrapure water three times and transferred to the volumetric flask, adding ultrapure water to dilute to 25 mL. The blank control solution was prepared in the same way. The sample solution was filtered through a 0.45 μm syringe filter. An appropriate dilution was performed in the final solution of 2% (*v*/*v*) HNO_3_ prior to analysis [35]. The standard solutions of As, Hg, Pb, and Cd were diluted to a series of concentrations by 2% (*v*/*v*) HNO_3._ All samples were analyzed in triplicate and quantified by the calibration curves.

### 3.5. Determination of N-acetyldopamine Oligomers 

*N*-acetyldopamine oligomers were qualitatively and quantitatively determined by our previously established UPLC-QTOF-MS/MS analysis [11]. UPLC was performed on a Waters ACQUITY UPLC ^TM^ system (Waters, Milford, MA, USA) furnished with a binary solvent delivery manager and an auto-sampler. The analytes were separated on a Waters ACQUITY HSS C18 column (100 mm × 2.1 mm, 1.8 µm) at 40 °C with a flow rate of 0.4 mL/min. The mobile-phase system consisted of (A) water containing 0.1% formic acid and (B) acetonitrile containing 0.1% formic acid. The gradient condition was optimized as follows: 15–50% B in 0–10 min; 50–95% B in 10–10.5 min; and 95% B in 10.5–12.5 min. The injection volume was set to 2 μL.

Mass spectrometry was performed on a Waters Q-TOF Synapt G2-S system (Waters MS Technologies, Manchester, UK) coupled with an electrospray ionization (ESI) source operating in positive mode, and the analytes were monitored in full scan mode. The parameters were as follows: the capillary voltage was 3 kV, the cone voltage was 40 V, the ion source temperature was 120 °C, the desolvent gas temperature was 450 °C, the desolvation gas flow rate was 800 L/h, the cone gas flow rate was 50 L/h, the collision-induced dissociation (CID) energy was 6 V, and the mass–charge ratio range was 100–1500 Da. All the MS data were processed by MassLynx 4.1 software (Waters Corporation, Milford, MA, USA).

One gram of the CP powder (through a 50-mesh sieve) was ultrasonically extracted with 10 mL of 80% (*v*/*v*) methanol at 25 °C for 60 min. The extract solution was centrifuged twice at 13,000 rpm for 15 min. The supernatant was collected and stored at 4 °C for analysis.

For the qualitative determination, the identities of all *N*-acetyldopamine oligomers were confirmed by comparing the mass spectra and the retention times of peaks with those of reference compounds and/or compounds tentatively assigned by matching empirical molecular formulas with those of published compounds and/or elucidating quasi-molecular ions and fragment ions by referring to available literature information. For the quantitative determination, the standard solutions of *N*-acetyldopamine dimer A and *N*-acetyldopamine dimer B were mixed, with a concentration of 100 ng/mL. *N*-acetyldopamine dimer A and *N*-acetyldopamine dimer B were quantified using a single-point external standard method, as the method had previously been validated [11]. The contents of side-chain isomers of dimers, trimers, side-chain isomers of trimers, tetramers, and pentamers were semi-quantified by using *N*-acetyldopamine dimer A as reference. The calculation formula was as follows:

Ar/Ai = Cr/Ci

where Ar represents the peak area of *N*-acetyldopamine dimer A, Ai represents the peak area of the analyte, and Cr and Ci represent the concentrations of *N*-acetyldopamine dimer A and the analyte, respectively.

### 3.6. Determination of Amino Acids

For the quantification of HAA, the sample pretreatment was as follows: 0.05 g of the CP powder (through an 80-mesh sieve) was added to the hydrolysis tube [36]. Then, 5 mL of 6 M hydrochloric acid was added to the hydrolysis tube, and the tube was filled with nitrogen, sealed, and placed in an oven at 110 °C for 22 h. Then, the hydrolyzate was taken out, and the volume was diluted to 100 mL with ultrapure water. Next, 1 mL of the diluted solution was evaporated to dryness in a rotary vacuum evaporator. Then, the residue was dissolved with 1 mL of water, and the solution was passed through a 0.22 μm hydrophilic PTFE membrane before being injected into the amino acid analyzer.

For the determination of FAA, 0.1 g of the CP powder (through an 80-mesh sieve) was extracted with 2 mL of ultrapure water overnight at 4 °C. The extract was centrifuged at 15,000 rpm for 20 min at 4 °C. The supernatant was transferred to a new centrifuge tube. Then, 600 μL of the supernatant was mixed with 600 μL of 4% sulfosalicylic acid, and the mixture was centrifuged at 15,000 rpm for 20 min at 4 °C. The solution was passed through a 0.22 μm hydrophilic PTFE membrane before being injected into the amino acid analyzer [36].

The amino acids of the CP samples were detected with an L-8900 automatic amino acid analyzer (Hitachi, Japan). HAA and FAA were qualitatively analyzed and quantified by comparing the retention times and peak areas of each amino acid standard [37,38].

### 3.7. Determination of DPPH and ABTS Radical Scavenging Rate

Both the DPPH and ABTS radicals are widely used as test radicals to assess the anti-oxidant activity and capacity of bioactive compounds. The DPPH assay was performed by referring to the literature [39,40]. First, the CP methanol extracts were reconstituted to concentrations ranging from 0.06 to 2.0 mg/mL, and 100 μL of each solution was added to a 96-well microplate. Then, 100 μL of a 200 μM DPPH methanol solution was added to each of the above samples. In addition, the blank group was set by mixing 100 μL of CP extracts with 100 μL of 80% methanol, and the control group was set by mixing 100 μL of 80% methanol with 100 μL of DPPH solution. Vitamin C (VC) was used as a positive control. The reaction mixtures were incubated in the dark for 30 min at room temperature. The absorbance was recorded at 517 nm using a microplate reader (Thermo Multiskan GO, Waltham, MA, USA). The DPPH**•** scavenging percentage was calculated by the following formula:

DPPH**•** scavenging (%) = [1 − (A_s_ − A_b_)/A_c_] × 100

where A_s_, A_b_, and A_c_ represent the absorbance of CP extract sample, blank, and control group, respectively. The IC_50_ value was calculated from the non-linear regression curve.

The ABTS assay was performed by referring to the literature [41,42]. Briefly, 5 mL of a 7 mM ABTS reserve solution was fully mixed with 5 mL of a 2.45 mM K_2_S_2_O_8_ solution and placed at room temperature in the dark for 14 h. The mixed solution was diluted with methanol to the absorbance of 0.7 ± 0.02 at 734 nm and was used as the working solution. Then, 160 μL of the working solution and 40 μL of the CP extract solution (0.03–1.0 mg/mL) were added to a 96-well microplate. After being incubated for 10 min in the dark at room temperature, the absorbance values of reaction mixtures were detected at 734 nm. In addition, 40 μL of an 80% methanol aqueous solution instead of the CP extract solution was used as the blank group, and 160 μL of methanol instead of the working solution was used as the control group. VC was used as a positive control. The ABTS^+^ scavenging percentage was calculated by the following formula:

ABTS^+^ scavenging (%) = [1 − (A_s_ − A_b_)/A_c_] × 100

where A_s_, A_b_, and A_c_ represent the absorbance of the CP extract sample, blank, and control group, respectively. The IC_50_ value was calculated from the non-linear regression curve.

### 3.8. Determination of Nitric Oxide (NO) 

The Griess reagent assay was used to detect the NO content in RAW264.7 macrophage cell supernatant to evaluate anti-inflammatory activity [43,44]. Briefly, RAW264.7 macrophages (1.6 × 10^5^ cells per well) were cultured in 96-well plates for 24 h at 37 °C in an atmosphere of 5% CO_2_. Then, the medium was removed. Then, 150 μL of fresh medium with the CP extract solution (2 mg/mL) and lipopolysaccharide (LPS, 1 μg/mL) were added to the cells for 24 h of treatment. The dexamethasone (DEX, 0.5 mg/mL) replacing the CP extract solution was used as a positive control. The medium without any treatment was used as a blank control. After that, 100 μL of the medium was collected and moved to a new 96-well plate. Next, 100 μL of Griess reagent was added to the well, and the mixture was incubated at 25 °C in the dark for 10 min. Absorbance was measured using a microplate reader (Thermo Multiskan GO, Waltham, MA, USA) at 540 nm, and the NO production was calculated based on a standard curve.

### 3.9. Statistical Analysis 

The data are presented as means ± standard deviations. Differences between groups were examined using the two-tailed Student’s *t*-test. A *p*-value < 0.05 was considered statistically significant. A Spearman’s correlation analysis was used to investigate the relationships between the contents of the multi-type components and the anti-oxidant/-inflammatory activities.

## 4. Conclusions

In this study, the effects of two washing methods on the quality and bioactivity of CP were systematically evaluated by comparing the chemical profiles and contents of *N*-acetyldopamine oligomers and the contents of amino acids, total ash, acid-insoluble ash, and heavy metals (As, Hg, Pb, and Cd) as well as the anti-oxidant and anti-inflammatory activities of the washed CP samples with those of the non-washed samples. Both pre-molting washing and post-molting washing could remarkably lower the residual silt and heavy metals of the CP samples. More importantly, pre-molting washing was found to be capable of significantly increasing the contents of *N*-acetyldopamine oligomers and amino acids because of the removal of residual silt from CP, whereas post-molting washing would cause the obvious loss of *N*-acetyldopamine oligomers and free amino acids, probably due to the additional contact of the medial side of CP with water. Furthermore, pre-molting-washed CP had significantly higher anti-oxidant and anti-inflammatory activities than post-molting-washed CP, probably owing to its higher contents of *N*-acetyldopamine oligomers and free amino acids. In conclusion, pre-molting washing can enhance the quality and bioactivity of CP and should be a superior alternative method for the primary processing of qualified CP.

## Figures and Tables

**Table 1:** Structural information of *N*-acetyldopamine oligomers identified in CP samples.

No.	*t_R_* (min)	Formula	Compound	Molecular Ion	Calcd. (*m*/*z*)	Exptl. (*m*/*z*)	Error (ppm)	Fragment Ion	Reference
1	2.79	C_20_H_22_N_2_O_6_	*N*-acetyldopamine dimer A *	[M+H]^+^	387.1556	387.1549	−1.8	328.1179,	[11,12,20,21]
								269.0809,	
								192.0653,	
								150.0550	
2	2.85	C_20_H_22_N_2_O_6_	*N*-acetyldopamine dimers	[M+H]^+^	387.1556	387.1550	−1.5	328.1178,	[11,12,20,21]
								269.0808,	
								192.0654,	
								150.0548	
3	3.01	C_20_H_22_N_2_O_6_	*N*-acetyldopamine dimer B *	[M+H]^+^	387.1556	387.1550	−1.5	328.1180,	[11,12,20,21]
								269.0805,	
								192.0653,	
								150.0545	
4	3.36	C_20_H_22_N_2_O_6_	*N*-acetyldopamine dimers	[M+H]^+^	387.1556	387.1551	−1.3	328.1178,	[11,12,20,21]
								269.0807,	
								192.0656,	
								150.0551	
5	3.60	C_20_H_20_N_2_O_6_	*N*-acetyldopamine dimers side-chain isomer	[M+H]^+^	385.1400	385.1394	−1.6	326.1022,	[11,12]
								192.0658,	
								150.0556	
6	4.24	C_30_H_31_N_3_O_9_	*N*-acetyldopamine trimers	[M+H]^+^	578.2139	578.2141	0.3	387.1544,	[11,12]
								385.1392,	
								192.0654	
7	4.34	C_30_H_31_N_3_O_9_	*N*-acetyldopamine trimers	[M+H]^+^	578.2139	578.2137	−0.3	387.1543,	[11,12]
								385.1392,	
								192.0653	
8	4.44	C_30_H_31_N_3_O_9_	*N*-acetyldopamine trimers	[M+H]^+^	578.2139	578.2139	0.0	387.1543,	[11,12]
								385.1390,	
								192.0654	
9	4.52	C_30_H_31_N_3_O_9_	*N*-acetyldopamine trimers	[M+H]^+^	578.2139	578.2137	−0.3	387.1549,	[11,12]
								385.1390,	
								192.0654	
10	4.64	C_30_H_31_N_3_O_9_	*N*-acetyldopamine trimers	[M+H]^+^	578.2139	578.2140	0.2	387.1547,	[11,12]
								385.1396,	
								192.0656	
11	4.75	C_30_H_31_N_3_O_9_	*N*-acetyldopamine trimers	[M+H]^+^	578.2139	578.2144	0.9	387.1550,	[11,12]
								385.1385,	
								192.0656	
12	4.92	C_30_H_31_N_3_O_9_	*N*-acetyldopamine trimers	[M+H]^+^	578.2139	578.2139	0.0	387.1544,	[11,12]
								385.1389,	
								192.0655	
13	5.01	C_30_H_29_N_3_O_9_	*N*-acetyldopamine trimers side-chain isomer	[M+H]^+^	576.1982	576.1982	0.0	517.1608,	[11,12]
								192.0656	
14	5.12	C_30_H_31_N_3_O_9_	*N*-acetyldopamine trimers	[M+H]^+^	578.2139	578.2137	−0.3	387.1537,	[11,12]
								192.0656	
15	5.20	C_30_H_29_N_3_O_9_	*N*-acetyldopamine trimers side-chain isomer	[M+H]^+^	576.1982	576.1980	−0.3	517.1620,	[11,12]
								192.0655	
16	5.28	C_40_H_40_N_4_O_12_	*N*-acetyldopamine tetramers	[M+H]^+^	769.2721	769.2724	0.4	576.1981,	[11,12]
								192.0657	
17	5.34	C_40_H_40_N_4_O_12_	*N*-acetyldopamine tetramers	[M+H]^+^	769.2721	769.2725	0.5	576.1978,	[11,12,22]
								192.0657	
18	5.44	C_40_H_40_N_4_O_12_	*N*-acetyldopamine tetramers	[M+H]^+^	769.2721	769.2720	−0.1	576.1976,	[11,12,22]
								192.0654	
19	5.49	C_40_H_40_N_4_O_12_	*N*-acetyldopamine tetramers	[M+H]^+^	769.2721	769.2719	−0.3	576.1972,	[11,12,22]
								192.0653	
20	5.56	C_40_H_40_N_4_O_12_	*N*-acetyldopamine tetramers	[M+H]^+^	769.2721	769.2719	−0.3	576.1973,	[11,12,22]
								192.0652	
21	5.63	C_40_H_40_N_4_O_12_	*N*-acetyldopamine tetramers	[M+H]^+^	769.2721	769.2715	−0.8	576.1976,	[11,12,22]
								192.0656	
22	5.73	C_40_H_40_N_4_O_12_	*N*-acetyldopamine tetramers	[M+H]^+^	769.2721	769.2725	0.5	576.1978,	[11,12,22]
								192.0655	
23	5.80	C_40_H_40_N_4_O_12_	*N*-acetyldopamine tetramers	[M+H]^+^	769.2721	769.2720	−0.1	576.1972,	[11,12,22]
								192.0654	
24	5.91	C_40_H_40_N_4_O_12_	*N*-acetyldopamine tetramers	[M+H]^+^	769.2721	769.2720	−0.1	576.1978,	[11,12,22]
								192.0655	
25	6.00	C_40_H_40_N_4_O_12_	*N*-acetyldopamine tetramers	[M+H]^+^	769.2721	769.2726	0.6	576.1980,	[11,12,22]
								192.0657	
26	6.14	C_50_H_49_N_5_O_15_	*N*-acetyldopamine pentamers	[M+H]^+^	960.3303	960.3306	0.3	767.2559,	[11,12]
								576.1974,	
								192.0654	
27	6.20.	C_50_H_49_N_5_O_15_	*N*-acetyldopamine pentamers	[M+H]^+^	960.3303	960.3310	0.7	767.2560,	[11,12]
								576.1970,	
								192.0654	
28	6.35	C_50_H_49_N_5_O_15_	*N*-acetyldopamine pentamers	[M+H]^+^	960.3303	960.3308	0.5	767.2560,	[11,12]
								576.1967,	
								192.0654	
29	6.41	C_50_H_49_N_5_O_15_	*N*-acetyldopamine pentamers	[M+H]^+^	960.3303	960.3315	1.2	767.2560,	[11,12]
								576.1974,	
								192.0654	
30	6.45	C_50_H_49_N_5_O_15_	*N*-acetyldopamine pentamers	[M+H]^+^	960.3303	960.3317	1.5	767.2570,	[11,12]
								576.1967,	
								192.0655	
31	6.63	C_50_H_49_N_5_O_15_	*N*-acetyldopamine pentamers	[M+H]^+^	960.3303	960.3312	0.9	767.2561,	[11,12]
								576.1967,	
								192.0656	
32	6.68	C_50_H_49_N_5_O_15_	*N*-acetyldopamine pentamers	[M+H]^+^	960.3303	960.3316	1.4	767.2559,	[11,12]
								576.1979,	
								192.0658	
33	6.85	C_50_H_49_N_5_O_15_	*N*-acetyldopamine pentamers	[M+H]^+^	960.3303	960.3303	0.0	767.2558,	[11,12]
								576.1978,	
								192.0656	
34	6.91	C_50_H_49_N_5_O_15_	*N*-acetyldopamine pentamers	[M+H]^+^	960.3303	960.3309	0.6	767.2559,	[11,12]
								576.1981,	
								192.0652	
35	6.99	C_30_H_29_N_3_O_9_	*N*-acetyldopamine trimers side-chain isomer	[M+H]^+^	576.1982	576.1981	−0.2	517.1597,	-
								192.0656	
36	7.18	C_30_H_29_N_3_O_9_	*N*-acetyldopamine trimers side-chain isomer	[M+H]^+^	576.1982	576.1979	−0.5	517.1615,192.0656	-
37	7.36	C_30_H_29_N_3_O_9_	*N*-acetyldopamine trimers side-chain isomer	[M+H]^+^	576.1982	576.1978	−0.7	192.0656	-
38	7.40	C_30_H_29_N_3_O_9_	*N*-acetyldopamine trimers side-chain isomer	[M+H]^+^	576.1982	576.1983	0.2	192.0654	-
39	7.58	C_30_H_29_N_3_O_9_	*N*-acetyldopamine trimers side-chain isomer	[M+H]^+^	576.1982	576.1976	−1.0	192.0656	-

* Confirmed with reference compound.

## Data Availability

Data are contained within the manuscript.
